# Gemcitabine-Induced Hemolytic Uremic Syndrome in Lung Cancer: A Case Report

**DOI:** 10.7759/cureus.20926

**Published:** 2022-01-04

**Authors:** Prashant Ahlawat, Monica Gupta, Prateek Upadhyay, Shivani Gupta, Amanjot Kaur

**Affiliations:** 1 General Medicine, Government Medical College & Hospital, Chandigarh, IND; 2 Anaesthesiology, Government Medical College & Hospital, Chandigarh, IND

**Keywords:** geriatrics, chemotherapy, lung cancer, hemolytic uremic syndrome, gemcitabine-induced hemolytic uremic syndrome

## Abstract

Gemcitabine is a broad-spectrum anti-metabolite drug that is widely used in the therapy of numerous advanced cancers such as pancreatic, breast, ovary, lung, and bladder cancer. Gemcitabine has been reported to cause hemolytic uremic syndrome (HUS), but the underlying mechanism is not elucidated. The outcome of gemcitabine-induced HUS is often poor and associated with high mortality. We present a case report of a patient who was on chemotherapy for lung cancer and presented with the concerns of decreased urine output and shortness of breath. He was investigated and found to have HUS*. *He was managed with plasmapheresis, which resulted in partial recovery. This case report describes HUS caused by gemcitabine in patients with lung carcinoma and the management implemented and also aims to highlight the importance of early and timely recognition and treatment to improve clinical outcomes in these patients.

## Introduction

Hemolytic uremic syndrome (HUS) is a principal subtype of thrombotic microangiopathic (TMA) syndromes that are characterized by common clinical and pathological features. Clinical features include microangiopathic hemolytic anemia (MAHA), thrombocytopenia, and target organ damage, i.e., acute kidney injury in HUS [1]. Pathological features include vascular thrombosis and endothelial damage. Endothelial damage in HUS is due to the overactivation of the alternate complement pathway [2]. Etiologically HUS can be classified into primary and secondary. Primary causes arise from either gene mutations or autoantibodies, with gene mutations being commoner. Genetic mutation is most commonly in factor H; other mutations include CD46, factors I and B, and thrombomodulin mutations. Autoantibodies against factor H can also develop [3]. Secondary causes include infections, drug-induced toxicity, pregnancy, autoimmune disorders, and disseminated malignancy. Common infectious agents to incite HUS include Shiga-toxin-producing *Escherichia coli*, which causes bloody diarrhea in children, HIV, and *Streptococcus pneumoniae*. The most common drugs related to secondary HUS include mitomycin, cisplatin, bleomycin, and, more recently, gemcitabine [4,5].

## Case presentation

A 65-year-old male presented to the hospital with chief concerns of swelling in the feet and a decreased urine output. He was a known case of lung carcinoma and was being managed chemotherapy for five months. As for his background history, he was diagnosed with lung cancer on a contrast-enhanced CT scan of the chest and was started on chemotherapy after biopsy confirmation, which was suggestive of squamous cell cancer - moderately differentiated. He was being managed on gemcitabine and carboplatin for five months. After completion of the fifth cycle, he noticed swelling in his feet, which gradually progressed and was associated with a decreased urine output. He presented to the hospital when he developed shortness of breath.

On clinical examination, he had a blood pressure of 160/90 mm Hg, although he was not a known case of hypertension, jugular venous pressure (JVP) was raised, and mild pallor and pedal edema was present. On chest auscultation, bilateral inspiratory coarse crepts were heard. Two-dimensional echocardiography was performed, which was within normal limits, and thus he was diagnosed with non-cardiogenic pulmonary edema.

**Figure 1:**
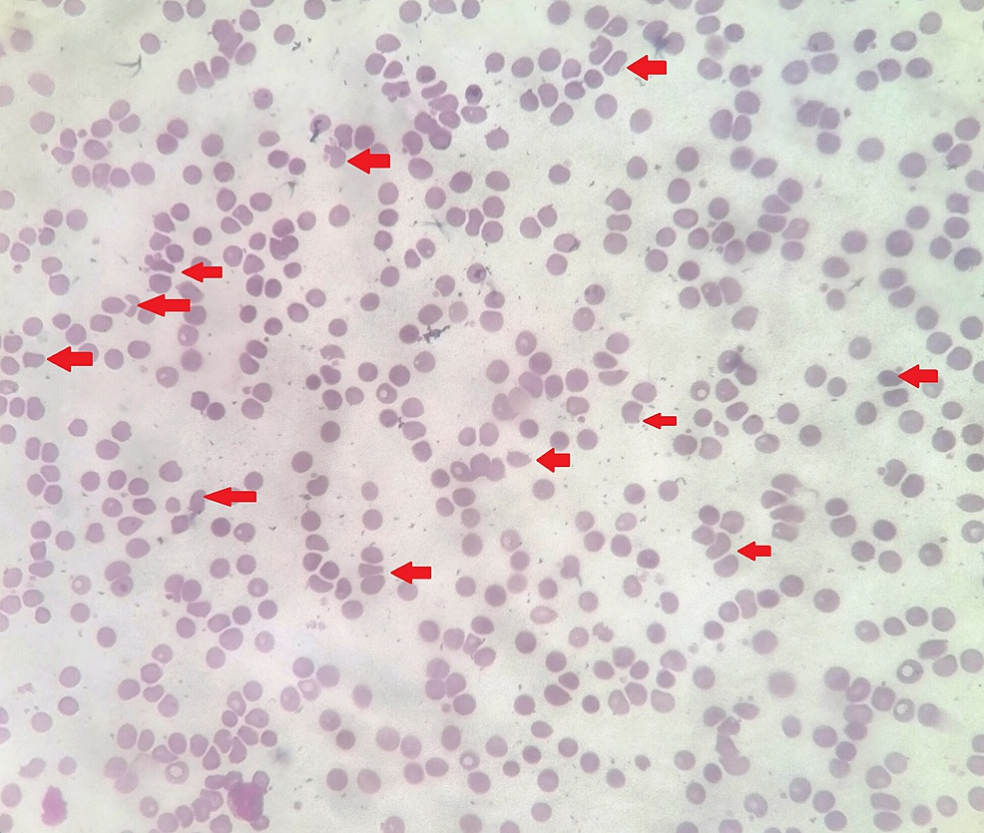
Peripheral blood film of the patient showing schistocytes, i.e., fragmented red cells (helmet cells) marked with arrows.

As per previous investigations, his cell counts, as well as kidney and liver function tests, were within normal limits. The routine investigations on this presentation revealed a hemoglobin concentration of 8.9 g/dL, deranged renal function tests (creatinine of 9.3), higher blood pressure records (>150/90 mm Hg at all times), and thrombocytopenia (platelet count of 79,000/mm^3^). On peripheral blood smear, schistocytes and fragmented red blood cells were present (Figure 1). Lactate dehydrogenase (LDH) was high (460 U/L) and haptoglobin was <20 mg/dL. The C3 and C4 levels were mildly decreased, coagulation profile was normal, and serum electrolytes and uric acid were within the normal range.

A thorough workup was performed to rule out tropical infections that are common causes of thrombocytopenia in developing countries like India, hence, dengue, malaria, scrub typhus, and leptospira. His HIV serology and hepatitis B and C serology were also negative. The patient did not receive any other drug besides the ongoing chemotherapy regimen. Ultrasonography of the abdomen presented a normal size of the kidneys with no parenchymal changes along with a normal-sized liver and spleen. Further workup for hemolysis was performed but indirect Coombs test (ICT) and direct Coombs test (DCT) came out as negative. Urine was negative for hemoglobin and was positive for albumin (1+). Blood culture, stool culture, and urine culture were sterile. The stool was examined for Shiga toxin and was negative.

Gemcitabine-induced HUS (GiHUS) was suspected and it was discontinued. The patient was managed with three cycles of hemodialysis to stabilization. He was then started on plasmapheresis following which his urine output and renal functions started to show an improving trend. After six cycles of plasmapheresis, the patient's creatinine level decreased to 3.3 mg/dL. He was able to maintain a urine output of about 700 mL, and platelet count also increased to 1,90,000/mm^3^. Plasmaphereses resulted in an observable, though mild improvement in kidney function. However, the patient declined further chemotherapy due to his poor general condition after HUS treatment. He was transferred to a nursing home for hospice care and has continued with outpatient palliative therapy in our hospital. The therapy is aiming to affirm the provision of a good quality of life. Palliative care is being offered through an amalgamation of symptomatic relief to patient along with psycho-social-spiritual support to the patient as well as the family. Physicians and caregivers are aiming to maintain the vital functioning of patients throughout the course of the disease.

## Discussion

Gemcitabine is a novel pyrimidine anti-metabolite class of drug that is used as a sole agent as well as in combination with other chemotherapy drugs. It is applicable in a wide range of carcinomas, such as pancreatic, non-small cell lung, breast, and ovarian and bladder carcinoma(s), in addition to lymphoma. Gemcitabine is cell cycle-specific in nature and targets the cell in its S phase. It undergoes phosphorylation intracellularly by the enzyme deoxycytidine kinase to gemcitabine biphosphate and further triphosphate. Gemcitabine biphosphate inhibits ribonucleotide reductase, whereas triphosphate is incorporated into DNA and inhibits DNA polymerase. It is usually well tolerated by the patient. The chief adverse effects occur due to its toxicity. This includes myelosuppression, nausea/vomiting, influenza-such as symptoms, rashes, and an elevation in liver enzymes [6].

In 1994, an introductory case of HUS was reported to occur in gemcitabine therapy during a phase II trial while aiming to devise a therapy for metastatic adenocarcinoma of the pancreas [7]. The incidence of GiHUS has been reported to be between 0.015% and 4% [8]. A strong dose-dependent relationship has not been established between the use of gemcitabine and the development of HUS, although cases of HUS developing even after a single dose of the drug have been reported [9]. HUS has been observed to occur even at a lower dose when gemcitabine is administered as a part of a combination therapy with other agents [10]. As in this case, it was used in combination with carboplatin in the chemotherapy regimen of the patient. The basic pathophysiology for GiHUS is indefinite. Many immune and non-immune theories have come up inconclusively [11]. The probable mechanism is direct endothelial damage to the kidneys, which results in platelet activation, release of ultra-large von Willebrand factor multimers, and activation of the clotting cascade [8]. In the literature, the median time between initiation of chemotherapy and GiHUS was 7.4 months and at a cumulative dose of 20,000 mg/m^2^ in a broad range of 2,450 to 48,000 mg/m^2^ with no clear dose-response relationship. Our patient began developing symptoms after five months of chemotherapy and at a cumulative dose of 12,044 mg/m^2^.

HUS is diagnosed clinically as a combination of Coomb’s negative hemolytic anemia (raised LDH, schistocytes on peripheral blood film, and decreased haptoglobin), thrombocytopenia, and organ damage, i.e., acute kidney injury [1,12]. The renal biopsy is a recommended modality to confirm the diagnosis of thrombotic microangiopathy; however, it lacks practicality since it is an invasive procedure with significant risks of hemorrhage. Hemorrhage can occur even with the transjugular approach that is used when low platelet count contraindicates the percutaneous approach. Hence, renal biopsy is not quite necessary if suggestive clinical and biological signs are present [8]. Microvascular damage to arterioles along with small arteries is seen in renal biopsy. These microvessels are shown to be occluded by eosinophilic hyaline thrombi, which are composed of fibrin and platelet aggregates. In our case, diagnosis of HUS was made clinically as supported by lab investigations. A renal biopsy was being planned for this patient, but it was decided to omit the procedure given risks of severe bleeding. Diagnosis of HUS may be delayed as the physician might consider anemia and thrombocytopenia as a consequence of chemotherapy and may continue treating the patient with gemcitabine along with colony-stimulating factors. Anemia is attributed to myelotoxicity of gemcitabine and due to GiHUS can be differentiated with the help of a reticulocyte count. A sudden decrease in hemoglobin, sudden renal failure, features of fluid overload such as peripheral edema and pulmonary congestion, and thrombocytopenia should alert the physician to consider the possibility of HUS and its timely stoppage. Renal function tests should be monitored along with trends of hemoglobin and platelet count while the patient is on this drug. Other causes of acute renal failure in cancer patients that deserve due consideration are sepsis, nephrotoxic agents, disseminated intravascular coagulation, cancer infiltration, and nephrocalcinosis. Sudden derangement should alert the physician, and a peripheral blood film and LDH level can confirm the diagnosis of HUS.

The initial step of management in a case of suspected GiHUS is the cessation of therapy [13]. Treatment for HUS has historically been plasmapheresis, and the goals are to stabilize the hemoglobin level, normalize the platelet count, and improve renal function [5]. However, the role of plasma exchange in GiHUS is uncertain. It has been observed in a review that when compared between patients who were treated with therapeutic plasma, 56% of those patients who were not treated with therapeutic plasma exchange recovered from gemcitabine-induced TMA. Contrarily, only 30% of patients who received plasma exchange recovered [13]. Eculizumab, a monoclonal antibody, acts as a terminal complement inhibitor that binds with high affinity to the human C5 complement and is a promising treatment for thrombotic microangiopathy. In a study on eculizumab, it was concluded that it led to normalized platelet counts at one week after the initiation of therapy in more than 50% of patients studied and a mean increase in estimated glomerular filtration rate of 32 mL/minute after 60 weeks [14]. Rituximab is also coming up as an effective drug that is well-tolerated by patients suffering from refractory HUS. It has been used successfully in two patients with gemcitabine-induced TMA refractory to commonly employed therapy of plasma exchange and steroids [15,16].

## Conclusions

GiHUS deserves a high level of clinical suspicion, and practicing physicians must be vigilant to diagnose it. A timely diagnosis can offer timely treatment to have the best clinical outcome. However, GiHUS is an often-unheeded condition, and the presentation gets attributed to other confounding variables. Treatment is not standardized at present, but various therapies have shown to be effective in management including plasma exchange. Palliative care should be commenced early for a holistic approach and to maintain the continuum of care. Newer drugs are showing promising results and might revolutionize the management of drug-induced HUS in the near future.

## References

[1] George JN, Nester CM (2014). Syndromes of thrombotic microangiopathy. N Engl J Med.

[2] Alasfar S, Alachkar N (2014). Atypical hemolytic uremic syndrome post-kidney transplantation: two case reports and review of the literature. Front Med (Lausanne).

[3] Furlan M, Robles R, Galbusera M (1998). Von Willebrand factor-cleaving protease in thrombotic thrombocytopenic purpura and the hemolytic-uremic syndrome. N Engl J Med.

[4] Kaplan BS, Ruebner RL, Spinale JM, Copelovitch L (2014). Current treatment of atypical hemolytic uremic syndrome. Intractable Rare Dis Res.

[5] Chandra D, Lawson S, Ramani P (2004). Atypical haemolytic uraemic syndrome as a complication of induction chemotherapy for acute lymphoblastic leukaemia. J Clin Pathol.

[6] Min YJ, Joo KR, Park NH, Yun TK, Nah YW, Nam CW, Park JH (2002). Gemcitabine therapy in patients with advanced pancreatic cancer. Korean J Intern Med.

[7] Richmond J, Gilbar P, Abro E (2013). Gemcitabine-induced thrombotic microangiopathy. Intern Med J.

[8] Izzedine H, Isnard-Bagnis C, Launay-Vacher V (2006). Gemcitabine-induced thrombotic microangiopathy: a systematic review. Nephrol Dial Transplant.

[9] Thomas JG, Sethi S, Norby SM (2011). Chronic thrombotic microangiopathy secondary to chemotherapy for urothelial carcinoma in a patient with a history of Wegener granulomatosis. Am J Kidney Dis.

[10] Zupancic M, Shah PC, Shah-Khan F (2007). Gemcitabine-associated thrombotic thrombocytopenic purpura. Lancet Oncol.

[11] Saif MW, Xyla V, Makrilia N, Bliziotis I, Syrigos K (2009). Thrombotic microangiopathy associated with gemcitabine: rare but real. Expert Opin Drug Saf.

[12] Izzedine H, Perazella MA (2015). Thrombotic microangiopathy, cancer, and cancer drugs. Am J Kidney Dis.

[13] Glezerman I, Kris MG, Miller V, Seshan S, Flombaum CD (2009). Gemcitabine nephrotoxicity and hemolytic uremic syndrome: report of 29 cases from a single institution. Clin Nephrol.

[14] Legendre CM, Licht C, Muus P (2013). Terminal complement inhibitor eculizumab in atypical hemolytic-uremic syndrome. N Engl J Med.

[15] Bharthuar A, Egloff L, Becker J, George M, Lohr JW, Deeb G, Iyer RV (2009). Rituximab-based therapy for gemcitabine-induced hemolytic uremic syndrome in a patient with metastatic pancreatic adenocarcinoma: a case report. Cancer Chemother Pharmacol.

[16] Willemsen AE, van Herpen CM, Wesseling P, Bult P, van Laarhoven HW (2011). Fatal thrombotic microangiopathy after a single dose of gemcitabine as fourth-line palliative treatment for metastasized ductal breast carcinoma. Acta Oncol.

